# Differential diagnosis of suspected neurogenic orthostatic hypotension in a patient with Parkinson disease

**DOI:** 10.1007/s10286-017-0427-5

**Published:** 2017-07-03

**Authors:** Mark Lew, Daniel Kremens

**Affiliations:** 1 Department of Neurology, University of Southern California, Los Angeles, CA USA; 2 Department of Neurology, Thomas Jefferson University, Philadelphia, PA USA

**Keywords:** Neurogenic orthostatic hypotension, Droxidopa, Parkinson disease

## Challenge question

What is the best approach to diagnose and start pharmacologic treatment for neurogenic orthostatic hypotension?

## Case presentation

Mr. B is a 65-year-old male diagnosed with mild Parkinson disease (PD) 5 years ago. His first symptom of PD was right upper extremity stiffness and tremor. He takes carbidopa/levodopa 25/100 mg three times daily (TID), carbidopa/levodopa extended-release 25/100 mg at night, rasagiline 1 mg once in the morning, and amantadine 100 mg twice daily (BID). He did not report symptoms of orthostatic hypotension (OH); however, he suffered an episode of syncope at night while returning to bed from the bathroom. He sustained no head trauma and briefly lost consciousness and awakened startled but not confused. He was immediately taken to the hospital emergency room where an electrocardiogram showed normal sinus rhythm and heart rate (HR) of 75 beats per minute (bpm). Blood tests were reportedly normal and he was sent home with instructions to follow-up with his neurologist.

Upon visiting his neurologist a few days later, Mr. B’s blood pressure (BP) was 158/84 mmHg with a HR of 72 bpm while supine, and 102/70 mmHg with a HR of 105 bpm immediately after standing. Due to the elevated standing HR, the neurologist rechecked standing BP and HR 2 min later and found that they were 88/60 mmHg and 107 bpm, respectively. The patient looked pale and felt unsteady. A complete blood count and basic metabolic panel were requested. Blood urea nitrogen and creatinine were elevated and consistent with dehydration. Hematocrit was 32% and hemoglobin was 10 gm/dL. Ferritin was low and red blood cells were microcytic. Review of the patient’s records showed that, 4 months prior, his hematocrit was 37% and his hemoglobin was 12 gm/dL. The patient was instructed to hydrate and was treated for microcytic ferropenic anemia with iron supplementation with ferrous sulfate tablets 325 mg TID [1]. A few weeks later, the patient reported no further symptoms of OH and no additional syncopal events.

## Expert commentary (Dr. Lew)

A patient with PD can have symptomatic OH that is not neurogenic in nature. Therefore, conducting a thorough patient history and doing pertinent laboratory evaluations are both extremely important. However, a patient with PD who previously has had non-neurogenic OH may later develop neurogenic OH (nOH). This, again, emphasizes the importance of a careful medical history and physical examination in all patients.

## Case continuation

A year later, the patient had another brief episode of syncope, again when standing, this time after dinner. In talking with the patient, his neurologist finds out that he was at his primary care practitioner (PCP) a week prior, and was found to have ankle edema. His PCP prescribed hydrochlorothiazide 25 mg taken orally once daily (QD), which the patient filled and took the day before his episode of syncope. Also during his PCP visit, the patient reported feeling “lightheaded” consistently upon standing. He works for the US Postal Service as a letter carrier and is on his feet for much of the day. He has new symptoms that include a decrease in energy and feeling “dizzy” by the end of the workday. His only other medical history includes urinary frequency and urgency related to a diagnosis of mild benign prostatic hyperplasia currently untreated. At this visit, his seated BP in the office was 135/70 mmHg with a HR of 72 bpm, and his standing BP was 78/60 mmHg with a HR of 82 bpm, when he reported feeling “woozy”. His BP lying at a 30-degree incline was 142/76 mmHg. His comorbidities, symptoms, orthostatic standing BP, and blunted heart rate response upon standing are indicative of nOH.

Initial treatment of his symptomatic nOH began by telling the patient to discontinue hidrochlorotiazide and explaining non-pharmacologic therapies, including increasing fluid intake to 2–3 L daily, salting food, and wearing compression garments (waist-high stockings and/or an abdominal binder). These measures, however, did not control his symptoms and, again, he developed ankle edema with the salt and fluid load. Efforts to achieve volume expansion also worsened urinary frequency.

As the conservative measures to treat his nOH were ineffective to control symptoms, the patient was started on droxidopa 100 mg TID and titrated upward every 3 days to 300 mg TID. He was instructed to take his last dose of droxidopa at least 3–4 h prior to bedtime. Treatment with droxidopa at this dosage resolved his clinical complaints of orthostasis and symptoms of nOH. To avoid possible supine hypertension (sHTN), he was also instructed to elevate the head of his bed by 30°.

## Expert commentary (Dr. Lew)

Initial treatment for symptomatic nOH should begin with non-pharmacologic therapy, including increasing fluid intake to 2–3 L daily, salting food, and wearing compression garments. Caution must be exercised in patients such as this with a prior medical history of edema manifesting poor tolerance to fluids and a high probability of sodium overload. Treatment with fludrocortisone, a mineralocorticoid that acts by retaining sodium and water, was therefore deemed inappropriate for this patient. As the patient has a history of benign prostate hyperplasia, a medication review should also be conducted, as alpha-blockers used to treat prostate problems may cause or aggravate nOH. Thus, for this patient, droxidopa would be an appropriate pharmacologic option to treat his symptomatic nOH.

## Expert commentary (Dr. Kremens)

I agree with Dr. Lew that the first approach is to use non-pharmacologic therapies, including increased hydration and salt consumption to help expand intravascular volume. I would also encourage physical maneuvers, such as tightening of the calf and buttock muscles, if the patient becomes symptomatic. He would also likely benefit from compression garments that include not only compression stockings but also an abdominal binder. I would also review whether he absolutely needed rasagiline, as this drug can contribute to lower BP. In this case, conservative measures were not sufficient nor well tolerated. Therefore, pharmacologic treatment is necessary.

Droxidopa would be an appropriate initial therapy for this patient, especially given the fact that he did not tolerate volume expansion due to pedal edema and urinary issues. Droxidopa is FDA-approved for the treatment of symptomatic nOH in PD and has demonstrated favorable tolerability [2, 3]. A potential side effect of droxidopa is sHTN. This can usually be managed by elevating the head of the bed by at least 30° and instructing the patient to avoid lying flat while on droxidopa. The patient could also be instructed to eat a small snack prior to bedtime, as this has been shown to relieve sHTN. Finally, a short-acting antihypertensive agent may be needed if sHTN persists.

Midodrine would also be an appropriate drug to consider in this case [4]. Midodrine, however, can significantly raise seated BP and the patient’s BP was already 135/70 mmHg. Midodrine can also cause scalp pruritus and possible urinary retention, which may be problematic given this patient’s history of prostate hypertrophy. Fludrocortisone is another option for treating nOH; however, in this case, it would likely worsen the sHTN and ankle edema. Similarly, it would be important to measure potassium regularly as hypokalemia is a frequent side effect of fludrocortisone. While droxidopa would be appropriate initial therapy for this patient, as a practical matter it should be noted that, at least in the US, some insurance companies might require that a patient have a trial of fludrocortisone or midodrine before covering the cost of droxidopa.

## Case conclusion

The patient was seen in follow-up 4 months later. He has had no further syncopal events, although he did complain of some ongoing lightheadedness upon standing. His droxidopa was titrated up to 400 mg TID and the patient now reports that his symptoms have improved.
